# Association between Polymorphisms in MicroRNAs and Risk of Urological Cancer: A Meta-Analysis Based on 17,019 Subjects

**DOI:** 10.3389/fphys.2017.00325

**Published:** 2017-05-19

**Authors:** Yu-Hui Wang, Han-Ning Hu, Hong Weng, Hao Chen, Chang-Liang Luo, Jia Ji, Chang-Qing Yin, Chun-Hui Yuan, Fu-Bing Wang

**Affiliations:** ^1^Department of Laboratory Medicine, Zhongnan Hospital of Wuhan UniversityWuhan, China; ^2^Center for Evidence-Based and Translational Medicine, Zhongnan Hospital of Wuhan UniversityWuhan, China; ^3^Department of Pathology, Zhongnan Hospital of Wuhan UniversityWuhan, China; ^4^Department of Laboratory Medicine, Wuhan Children's Hospital, Huazhong University of Science and TechnologyWuhan, China

**Keywords:** microRNA-196a2, microRNA-146a, microRNA-499, polymorphism, urological cancers, meta-analysis

## Abstract

Accumulating evidence has demonstrated that some single nucleotide polymorphisms (SNPs) existing in miRNAs correlate with the susceptibility to urological cancers. However, a clear consensus still not reached due to the limited statistical power in individual study. Thus, we concluded a meta-analysis to systematically evaluate the association between microRNA SNPs and urological cancer risk. Eligible studies were collected from PubMed, Embase, Web of Science, and CNKI databases. Pooled odds ratio (OR) and corresponding 95% confidence interval (95% CI) were calculated to assess the strength of the relationships between three SNPs (miR-196a2, C>T rs11614913; miR-146a, G>C rs2910164; and miR-499, A>G rs3746444) and the risk of urological cancers. In addition, the stability of our analysis was evaluated by publication bias, sensitivity and heterogeneity analysis. Overall, a total of 17,019 subjects from 14 studies were included in this meta-analysis. We found that CT (miR-196a2, C>T rs11614913) was a risk factor for renal cell carcinoma (CT vs. CC: OR = 1.72, 95%CI = 1.05–2.80, *P* = 0.03, *I*^2^ = 66%), especially in Asian population (CT vs. CC: OR = 1.17, 95%CI = 1.04–1.32, *P* < 0.01, *I*^2^ = 0%). miR-146a G>C rs2910164 was a protective factor of urological cancers (C vs. G: OR = 0.87, 95%CI = 0.81–0.93, *P* < 0.01, *I*^2^ = 0%), especially for bladder cancer. miR-499 A>G rs3746444 was correlated with an increased risk of urological cancers, specifically in Asian population. In conclusion, our meta-analysis suggests that polymorphisms in microRNAs, miR-196a2, C>T rs11614913, miR-146a G>C rs2910164 and miR-499 A>G rs3746444, may be associated with the development of urological cancers and the risks mainly exist in Asian populations.

## Introduction

Urological cancers, which consist of prostate cancer (PCa), bladder cancer (BC), and renal cell cancer (RCC) are common malignancies with increasing incidence and mortality worldwide (Torre et al., 2015). According to the most recent cancer statistics, urological cancers constitute more than 33% of all cancers in the United States in 2016 (Siegel et al., 2016). PCa, specifically, is the most common cancer in males accounting for 21% of new diagnoses with about 180, 890 new cases (Siegel et al., 2016). Aside from environmental factors, like tobacco exposure, genetic predisposition also involved in the occurrence and development of urological cancers (Mikhaylova et al., 2012; Solomon et al., 2013; Attard et al., 2016). Single nucleotide polymorphisms (SNPs) are the most common form of genetic variation in the human genome and widely implicated in cancer occurrence, development, and treatment response (Sachidanandam et al., 2001; Zheng et al., 2008; Rothman et al., 2010; Laurie et al., 2012).

micro RNAs (miRNAs) are a group of small non-coding RNAs with ~22 nucleotides in length that play key roles in vital biological processes such as cell differentiation, metabolism, intracellular signaling, immunity, and cell movement (Bartel, 2004). Over the past decade, accumulating evidence has demonstrated that some SNPs occurred in miRNAs can alter miRNA physiological function such as interaction with the translation of messenger RNA (mRNA) to regulate the expression level of target genes, and thus showed great potential for risk assessment, diagnosis and prognosis evaluation in different cancers (Ameres and Zamore, 2013). Hu et al. identified the early-stage NSCLC patients with an SNP, miR-30c-1 rs928508, had a better survival and the prognostic predictive value of miR-30c-1 rs928508 risk score was significantly increased (Hu et al., 2011).

Recently, SNPs in miRNAs (miR-SNPs) have been confirmed to be associated with urological cancer risks in different studies (Chirila et al., 2015; Filella and Foj, 2016). Nikolic et al. provided the evidence that miR-27a rs895819 was associated with the presence of distant metastasis among PCa patients (Nikolic et al., 2015). miR-196a2 C>T (rs11614913), miR-146a G>C (rs2910164), and miR-499 A>G (rs3746444) have been widely studied to evaluate the correlation with the risk of urological cancers. However, the research results cannot reach consensus. For miR-196a2 C>T (rs11614913), Mittal et al. found that there was no association between the individuals carrying the variant genotype of the miRNA and bladder cancer risk (Mittal et al., 2011). On the contrary, Deng et al. suggested mir-196a2 rs11614913 was associated with a significantly decreased risk of bladder cancer (Deng et al., 2015). Therefore, we performed a meta-analysis of the related publications to systematically evaluate the association between these miR-SNPs and cancer risk, which may with the potential to be used as clinical parameters for assessing the risk of occurrence, development, as well as for response to treatment of urological cancers (Ryan et al., 2010; Eeles et al., 2014; Shukla et al., 2016).

## Materials and methods

### Literature search strategy

The databases including PubMed, Embase, Web of Science and CNKI were comprehensively searched to obtain literatures that reported the association between urological cancers and miRNA polymorphisms and that published in English or Chinese up to November 13, 2016. The search terms were as follows: “bladder cancer,” “testis cancer,” “prostate cancer,” “kidney cancer,” “microRNA,” “miRNAs,” “polymorphism,” and “polymorphisms.” In addition, to identify the additional relevant literatures, the references of searched studies were also examined carefully and the combined phrases were used.

### Inclusion and exclusion criteria

If the obtained studies fulfilled the following criteria, they were identified eligible: (1) case-control design; (2) research on the association between polymorphisms in microRNAs and risk of urological cancer; (3) sufficient published genotype frequencies data to estimate the odds ratio (OR) and 95% confidence interval (CI). The exclusion criteria included: (1) the genotype frequencies data was unavailable; (2) animal model research; (3) review articles, case reports, meta-analysis; (4) overlapping publications (the studies with more subjects or recently published were included).

### Data extraction

There were two independent researchers (Ji and Luo) to extract the data from all included studies for analysis, including first author, year of publication, country, ethnicity (classified as either Asian or non-Asian), source of controls, number of different genotypes, Hardy-Weinberg equilibrium (HWE) for controls, genotyping method and cancer type. The third reviewer (Wang) joined the discussion if some discrepancies existed.

### Quality assessment

The quality scoring criteria was modified from previous literatures and the score ranged from 0 point to 9 points (Table 1; Niu et al., 2015a). Two independent investigators (Wang and Ji) evaluated the quality of articles according to the modified criteria. A study with a score of ≥6 was defined as high quality, meantime one with a score <6 was low quality.

**Table 1:** **The criteria for quality assessment**.

**Criteria**	**Score**
**REPRESENTATIVENESS OF CASES**	
Continuous collection and representative cases within clearly defined limits	2
With potential selection bias	1
Not described	0
**SOURCE OF CONTROLS**	
Population-based	2
Hospital-based	1
Not described	0
**HARDY-WEINBERG EQUILIBRIUM IN CONTROLS**	
Hardy-Weinberg equilibrium	2
Hardy-Weinberg disequilibrium	1
**GENOTYPING EXAMINATION**	
Genotyping done under “blinded” condition	1
Unblinded done or not mentioned	0
**STATISTICAL METHODS**	
Appropriate statistics and adjustment for confounders	2
Appropriate statistics but without adjustment for confounders	1
Inappropriate statistics used	0

### Statistical analysis

To assess the strength of the association between the three polymorphisms in miRNA and urological cancer risks, the odds ratios (ORs) with corresponding 95% confidence intervals (CIs) were served as effect size. For the miR-196a2 C>T (rs11614913) polymorphism, the allelic (T vs. C), heterozygous (CT vs. CC), homozygous (TT vs. CC), dominant (CT+TT vs. CC) and recessive (TT vs. CC+CT) genetic models were used to obtain pooled ORs. These models were also applied to assess the miR-146a G>C (rs2910164), and miR-499 A>G (rs3746444) polymorphisms. The subgroup analysis was performed according to cancer type, ethnicity, source of controls and HWE status of controls. The Cochran's Q statistic and *I*^2^ test were used to access the heterogeneity between different studies (Higgins, 2008). Heterogeneity was acceptable when the *P*-value was more than 0.10 and *I*^2^ was <50%, and a fixed-effects model (the Mantel-Haenszel method) was used. In contrast, ORs were calculated by the random effects model (DerSimonian and Laird method; Mantel and Haenszel, 1959; Higgins et al., 2009). To evaluate the robustness of the results, we further performed the sensitivity analysis. Publication bias was conducted using Egger's linear regression and Begg's funnel plots (Hayashino et al., 2005). Statistical analysis of the data was calculated using the STATA version 12.0 (Stata Corp, College Station, TX, USA) with two-sided *P*-value. *P*-value was < 0.05 was considered significant.

## Results

### Study characteristics

**Figure 1:**
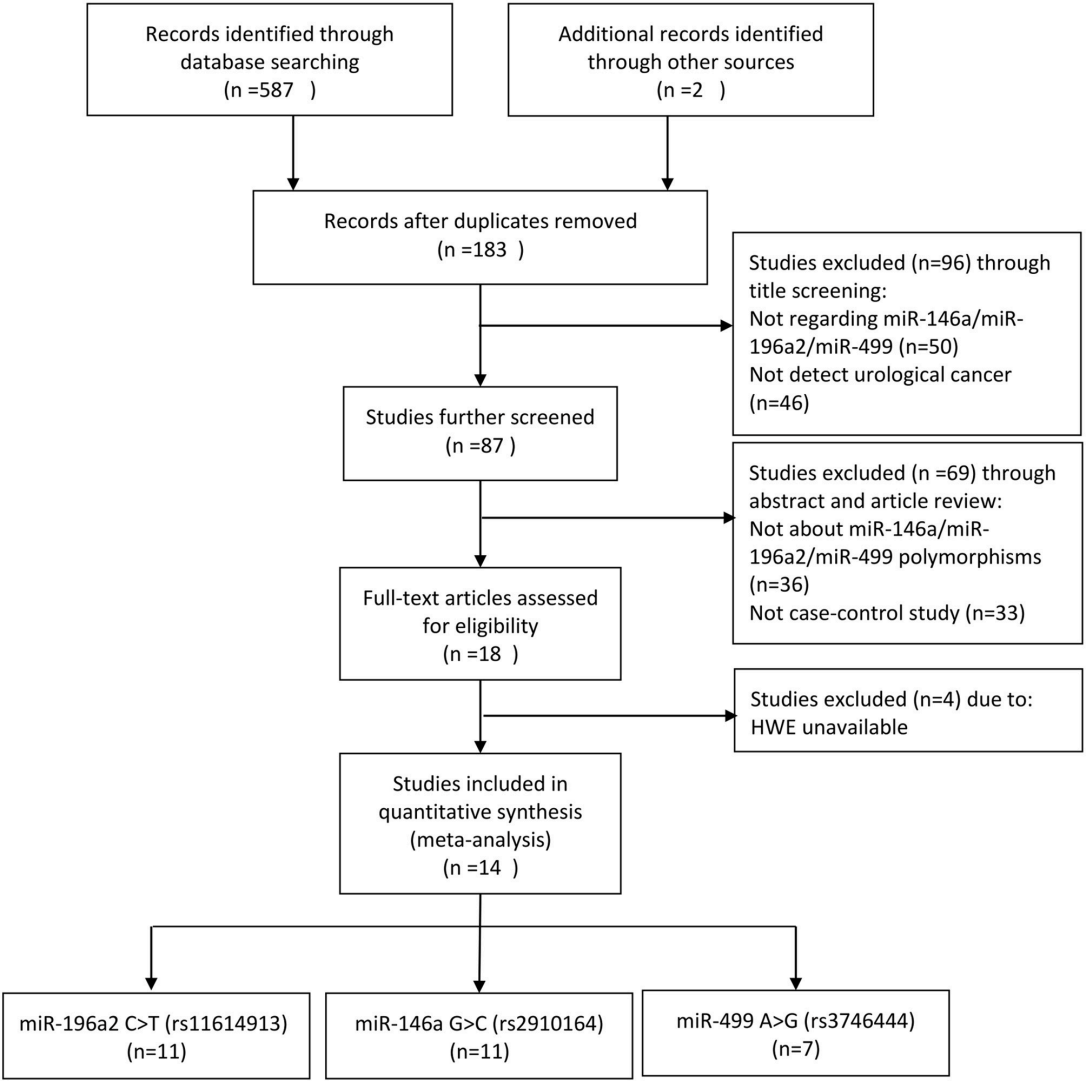
**Flow diagram of the study selection process**.

In the beginning, a total of 589 relevant publications was identified after a systematic literature search using our search strategy (Figure 1). 571 of 589 publications were excluded in the step of duplicate removed, title and abstract screening, and article review. Then, 18 articles were remained to further access the eligibility by reviewing the full article. Four articles were excluded due to lacking HWE information. Eventually, 14 eligible articles (29 studies for polymorphisms analysis of individual miRNA) were selected for our present meta-analysis. Eleven studies focused on miR-196a2 C>T (rs11614913) (Horikawa et al., 2008; Yang et al., 2008; George et al., 2011; Mittal et al., 2011; Du et al., 2014; Deng et al., 2015; Nikolic et al., 2015; Hashemi et al., 2016; Toraih et al., 2016a,b); 11 studies focused on miR-146a G>C (rs2910164) (Horikawa et al., 2008; Yang et al., 2008; Xu et al., 2010; George et al., 2011; Mittal et al., 2011; Wang et al., 2012; Du et al., 2014; Nikolic et al., 2014; Deng et al., 2015; Huang et al., 2015; Hashemi et al., 2016) and seven studies focused on miR-499 A>G (rs3746444) (George et al., 2011; Mittal et al., 2011; Du et al., 2014; Deng et al., 2015; Nikolic et al., 2015; Hashemi et al., 2016; Toraih et al., 2016a). Five articles (George et al., 2011; Mittal et al., 2011; Du et al., 2014; Deng et al., 2015; Hashemi et al., 2016) investigated polymorphisms of these three miRNAs. miR-196a2 C>T (rs11614913) and miR-146a G>C (rs2910164) were simultaneously detected in four articles (Horikawa et al., 2008; Yang et al., 2008; Nikolic et al., 2015; Toraih et al., 2016a). Table 2 presented the characteristics of the included studies. 10 studies focused on the association of miRNA polymorphisms and bladder cancer (Yang et al., 2008; Mittal et al., 2011; Wang et al., 2012; Deng et al., 2015; Toraih et al., 2016a) or prostate cancer (Xu et al., 2010; Nikolic et al., 2014, 2015; Hashemi et al., 2016), respectively. There were nine studies on renal cell cancer (Horikawa et al., 2008; Du et al., 2014; Huang et al., 2015; Toraih et al., 2016a,b). A large portion of studies were performed in Asian and the control was chosen from healthy population. As for the genotyping method, polymerase chain reaction-restriction fragment length polymorphism (PCR-RFLP) was used in 13 out of 29 studies. Some emerging tools, such as Taqman and SNPlex were applied to other studies. Within the genotype distribution in the controls, the value of HWE was either extracted in the articles directly or calculated using the data of controls. And only three studies (George et al., 2011; Mittal et al., 2011; Hashemi et al., 2016) deviated from HWE. The quality score of studies was also showed in Table 2.

**Table 2:** **Main characteristics of included studies on microRNA polymorphisms and urological cancer risk**.

**First author**	**Year**	**Country**	**Ethnicity**	**Source of controls**	**Case**	**Control**	**Genotype distribution**						**Genotyping methods**	***P* for HWE^a^**	**Cancer type**	**Quality score**
							**Case**			**Control**						
**miR-196a2 C>T (rs11614913)**																
							**CC**	**CT**	**TT**	**CC**	**CT**	**TT**				
Horikawa	2008	America	Non-Asian	Population	276	277	105	126	45	101	117	59	SNPlex	0.02	Renal cell carcinoma	8
Yang	2008	America	Non-Asian	Population	736	731	255	348	133	257	117	132	SNPlex	0.33	Bladder cancer	8
George	2011	Indian	Asian	Population	159	230	55	101	3	106	342	10	PCR-RFLP	< 0.01	Prostate cancer	7
Mittal	2011	Indian	Asian	Hospital	212	250	76	131	5	109	114	14	PCR-RFLP	< 0.01	Bladder cancer	7
Du	2014	China	Asian	Population	353	362	43	189	121	74	127	109	TaqMan	0.97	Renal cell carcinoma	8
Deng	2015	China	Asian	Population	159	298	41	66	52	56	179	76	PCR-RFLP	0.04	Bladder cancer	7
Nikolic	2015	Serbia	Non-Asian	Population	355	312	150	161	40	121	166	41	PCR-RFLP	0.73	Prostate cancer	8
Hashemi	2016	Iran	Asian	Hospital	169	182	64	88	17	77	147	12	T-ARMS-PCR	0.02	Prostate cancer	7
Toraih a	2016	Egypt	Non-Asian	Population	14	100	3	10	1	55	93	10	TaqMan	0.221	Renal cell carcinoma	8
Toraih a	2016	Egypt	Non-Asian	Population	13	100	7	3	3	55	35	10	TaqMan	0.221	Bladder cancer	8
Toraih b	2016	Egypt	Non-Asian	Population	65	150	23	31	11	80	53	17	TaqMan	0.082	Renal cell carcinoma	6
**miR-146a G>C (rs2910164)**																
							**GG**	**GC**	**CC**	**GG**	**GC**	**CC**				
Horikawa	2008	America	Non-Asian	Population	261	235	144	103	14	126	94	15	SNPlex	0.65	Renal cell carcinoma	8
Yang	2008	America	Non-Asian	Population	691	674	414	242	35	385	258	31	SNPlex	0.14	Bladder cancer	7
Xu	2010	China	Asian	Population	215	280	68	135	48	54	150	76	PCR-RFLP	0.19	Prostate cancer	7
George	2011	Indian	Asian	Population	159	230	4	79	76	7	107	116	PCR-RFLP	< 0.01	Prostate cancer	7
Mittal	2011	Indian	Asian	Hospital	212	250	127	79	6	135	108	7	PCR-RFLP	0.01	Bladder cancer	8
Wang	2012	China	Asian	Hospital	1,017	1,179	369	456	192	340	571	268	Taqman	0.34	Bladder cancer	6
Du	2014	China	Asian	Population	353	362	68	167	118	57	190	115	Taqman	0.14	Renal cell carcinoma	8
Nikolic	2014	Serbia	Non-Asian	Population	286	199	184	90	12	129	63	7	Taqman	0.84	Prostate cancer	7
Deng	2015	China	Asian	Population	159	298	26	73	60	32	154	112	PCR-RFLP	0.05	Bladder cancer	7
Huang	2015	China	Asian	Hospital	421	432	80	236	105	69	234	129	ABI sequencing system	0.03	Renal cell carcinoma	7
Hashemi	2016	Iran	Asian	Hospital	169	182	25	131	13	24	147	11	T-ARMS-PCR	< 0.01	Prostate cancer	6
**miR-499 A>G (rs3746444)**																
							**AA**	**AG**	**GG**	**AA**	**AG**	**GG**				
George	2011	Indian	Asian	Population	159	230	48	98	13	104	92	34	PCR-RFLP	0.07	Prostate cancer	8
Mittal	2011	Indian	Asian	Hospital	212	250	95	92	25	121	94	35	PCR-RFLP	0.02	Bladder cancer	8
Du	2014	China	Asian	Population	354	362	251	94	9	255	96	11	Taqman	0.59	Renal cell carcinoma	8
Deng	2015	China	Asian	Population	159	298	107	45	7	216	68	14	PCR-RFLP	0.01	Bladder cancer	7
Nikolic	2015	Serbia	Non-Asian	Population	355	307	190	147	18	180	110	17	PCR-RFLP	0.97	Prostate cancer	8
Hashemi	2016	Iran	Asian	Hospital	169	182	62	82	25	85	64	33	PCR-RFLP	< 0.01	Prostate cancer	6
Toraih b	2016	Egypt	Non-Asian	Population	65	150	6	17	42	57	66	27	TaqMan	0.307	Renal cell carcinoma	6

*Population, Population controls; Hospital, Hospital controls; PCR-RFLP, polymerase chain reaction–restriction fragment length polymorphism; T-ARMS-PCR, tetra-primer amplification refractory mutation system polymerase chain reaction; HWE, Hardy-Weinberg equilibrium*.

### Quantitative data synthesis

There were 11 eligible studies with 2,511 cases and 2,992 controls that focused on the association of miR-196a2 rs11614913 C>T and urological cancer risks. Overall, no significant association was revealed in the pooled results under any genetic model statistically. Table 3 presented the detailed results of the meta-analysis. The heterogeneity was significantly reduced by stratified analysis. Through stratified analyses by cancer type, heterozygote CT had an effect of increasing the risk of renal cell carcinoma compared with homozygote CC (OR = 1.72, 95%CI = 1.05–2.80, *P* = 0.03 *I*^2^ = 66%). However, miR-196a2 rs11614913 C>T showed no significant correlation with risks in prostate cancer and bladder cancer. In ethnicity subgroup analysis, significantly increased cancer risks were observed in Asian populations for allele genetic model (OR = 1.17, 95%CI = 1.04–1.32, *P* < 0.01, *I*^2^ = 0%). For subgroup analysis based on source of controls and HWE status of controls, no significant association was found. Sensitivity analysis showed that none of the studies leaded to change the global ORs, indicating the robustness and stable of the results in this meta-analysis. Begg's funnel plot and Egger's test showed that no obvious publication bias existed in the eligible literatures (T vs. C: *P* = 0.08; TC vs. CC: *P* = 0.33; TT vs. CC: *P* = 0.68; TC+ TT vs. CC: *P* = 0.15; TT vs. CC+ TC: *P* = 0.78).

**Table 3:** **Summary ORs and 95% CI of microRNA polymorphisms and urological cancer risk**.

**Variables**	**N**	**T vs. C**			**CT vs. CC**			**TT vs. CC**			**CT+TT vs. CC**			**TT vs. CC+CT**		
mir-196a2		OR (95%CI)	P	*I*^2^(%)	OR (95%CI)	P	*I*^2^(%)	OR (95%CI)	P	*I*^2^(%)	OR (95%CI)	P	*I*^2^(%)	OR (95%CI)	P	*I*^2^ (%)
Total	11	1.07 [0.99, 1.16]	0.08	43	1.21 [0.96, 1.54]	0.11	67	1.06 [0.89, 1.26]	0.5	45	1.21 [0.98, 1.50]	0.08	64	1.03 [0.89, 1.20]	0.67	36
**CANCER TYPE**																
Prostate cancer	3	1.04 [0.89, 1.22]	0.6	47	1.18 [0.80, 1.74]	0.41	66	0.93 [0.62, 1.38]	0.7	35	1.16 [0.80, 1.69]	0.43	65	0.92 [0.63, 1.33]	0.65	41
Bladder cancer	4	1.03 [0.92, 1.16]	0.6	0	0.94 [0.62, 1.44]	0.78	71	0.98 [0.76, 1.25]	0.85	0	1.03 [0.87, 1.22]	0.75	49	1.08 [0.70, 1.69]	0.72	56
Renal cell carcinoma	4	1.27 [0.92, 1.76]	0.15	73	**1.72 [1.05, 2.80]**	**0.03**	**66**	1.42 [0.73, 2.76]	0.3	70	1.69 [0.99, 2.89]	0.06	74	1.04 [0.82, 1.32]	0.74	39
**ETHNICITY**																
Asian	5	**1.17 [1.04, 1.32]**	**<0.01**	**0**	1.25 [0.84, 1.85]	0.27	76	1.13 [0.69, 1.86]	0.63	56	1.28 [0.93, 1.78]	0.13	68	1.17 [0.94, 1.47]	0.17	48
Non-Asian	6	1.06 [0.87, 1.29]	0.55	56	1.14 [0.85, 1.53]	0.38	54	0.96 [0.78, 1.20]	0.74	30	1.12 [0.85, 1.48]	0.42	57	0.94 [0.77, 1.14]	0.54	11
**DESIGN**																
Hospital	2	1.14 [0.93, 1.41]	0.2	0	1.32 [0.99, 1.76]	0.06	0	0.98 [0.30, 3.18]	0.98	68	1.30 [0.98, 1.73]	0.07	0	0.84 [0.22, 3.18]	0.8	77
Population	9	1.10 [0.95, 1.26]	0.19	53	1.20 [0.90, 1.61]	0.22	72	1.06 [0.89, 1.27]	0.52	47	1.20 [0.92, 1.57]	0.17	70	1.04 [0.89, 1.21]	0.62	30
**HWE**																
HWE-Yes	6	1.17 [0.96, 1.44]	0.12	62	1.34 [0.93, 1.95]	0.12	69	1.32 [0.89, 1.95]	0.16	54	1.36 [0.95, 1.96]	0.09	71	1.07 [0.89, 1.28]	0.46	0
HWE-No	5	1.05 [0.93, 1.19]	0.46	5	1.12 [0.79, 1.59]	0.53	72	0.85 [0.63, 1.15]	0.29	11	1.12 [0.84, 1.49]	0.46	61	0.89 [0.54, 1.46]	0.64	63
		C vs. G			GC vs. GG			CC vs. GG			CC+GC vs. GG			CC vs. GC+GG		
mir-146a		OR (95%CI)	P	*I*^2^(%)	OR (95%CI)	P	*I*^2^(%)	OR (95%CI)	P	*I*^2^(%)	OR (95%CI)	P	*I*^2^(%)	OR (95%CI)	P	*I*^2^(%)
Total	11	**0.87 [0.81,0.93]**	**<0.01**	**0**	**0.81 [0.73,0. 90]**	**<0.01**	**0**	**0.73 [0.63,0.85]**	**<0.01**	**0**	**0.80 [0.72,0.88]**	**<0.01**	**0**	**0.87 [0.77,0.98]**	**0.02**	**0**
**CANCER TYPE**																
Prostate cancer	4	0.89 [0.77,1.03]	0.11	30	0.87 [0.68, 1.12]	0.29	0	0.71 [0.49, 1.05]	0.08	32	0.85 [0.66, 1.08]	0.18	1	0.83 [0.64,1.07]	0.15	14
Bladder cancer	4	**0.84 [0.77, 0.92]**	**<0.01**	**0**	**0.78 [0.68, 0.89]**	**<0.01**	**0**	**0.72 [0.59, 0.87]**	**<0.01**	**0**	**0.77 [0.68, 0.88]**	**<0.01**	**5**	0.87 [0.73, 1.03]	0.1	0
Renal cell carcinoma	3	0.91 [0.80, 1.03]	0.13	0	0.86 [0.69, 1.07]	0.17	0	0.78 [0.59, 1.03]	0.08	0	0.85 [0.68, 1.04]	0.12	0	0.91 [0.74, 1.12]	0.35	8
**ETHNICITY**																
Asian	8	**0.85 [0.78, 0.91]**	**<0.01**	**0**	**0.76 [0.66, 0.86]**	**<0.01**	**0**	**0.69 [0.59, 0.82]**	**<0.01**	**0**	**0.74 [0.65, 0.83]**	**<0.01**	**0**	**0.85 [0.75, 0.97]**	**0.01**	**0**
Non-Asian	3	0.95 [0.83, 1.09]	0.5	0	0.91 [0.77, 1.09]	0.31	0	1.01 [0.69, 1.48]	0.97	0	0.93 [0.78, 1.09]	0.36	0	1.04 [0.71, 1.52]	0.83	0
**DESIGN**																
Hospital	4	**0.83 [0.76, 0.91]**	**<0.01**	**0**	**0.77 [0.66, 0.90]**	**<0.01**	**0**	**0.69 [0.57, 0.84]**	**<0.01**	**0**	**0.75 [0.65, 0.86]**	**<0.01**	**0**	**0.81 [0.68, 0.95]**	**0.01**	**0**
Population	7	0.91 [0.83, 1.00]	0.05	0	0.85 [0.73, 0.98]	0.02	0	0.79 [0.63, 0.99]	0.05	0	0.85 [0.74, 0.98]	0.02	0	0.94 [0.79, 1.11]	0.45	0
**HWE**																
HWE-Yes	9	**0.86 [0.80, 0.93]**	**<0.01**	**16**	**0.80 [0.72, 0.90]**	**<0.01**	**0**	**0.72 [0.61, 0.85]**	**<0.01**	**8**	**0.79 [0.71, 0.89]**	**<0.01**	**14**	**0.87 [0.76, 1.00]**	**0.06**	**11**
HWE-No	2	0.89 [0.78, 1.02]	0.09	0	0.84 [0.66, 1.07]	0.16	0	0.79 [0.56, 1.12]	0.19	0	0.82 [0.65, 1.04]	0.1	0	0.86 [0.68, 1.08]	0.18	0
		G vs. A			AG vs. AA			GG vs. AA			GG+AG vs. AA			GG vs. AG+AA		
mir-499		OR(95%CI)	P	*I*^2^(%)	OR (95%CI)	P	*I*^2^(%)	OR(95%CI)	P	*I*^2^(%)	OR(95%CI)	P	*I*^2^(%)	OR(95%CI)	P	*I*^2^(%)
Total	7	1.33 [0.98, 1.81]	0.06	85	**1.37 [1.18, 1.60]**	**<0.01**	**49**	1.33 [0.71, 2.48]	0.38	79	**1.43 [1.09, 1.88]**	**<0.01**	**67**	1.10 [0.55, 2.23]	0.78	87
**CANCER TYPE**																
Prostate cancer	3	1.16 [0.98, 1.36]	0.08	0	**1.68 [1.17, 2.41]**	**<0.01**	**58**	0.96 [0.65, 1.42]	0.84	0	**1.45 [1.17, 1.80]**	**<0.01**	**26**	0.72 [0.50, 1.03]	0.07	0
Bladder cancer	2	1.09 [0.87, 1.35]	0.46	0	1.28 [0.96, 1.72]	0.09	0	0.94 [0.57, 1.53]	0.79	0	1.21 [0.92, 1.59]	0.18	0	0.85 [0.53, 1.36]	0.5	0
Renal cell carcinoma	2	2.22[0.42,11.65]	0.35	97	1.37 [0.59, 3.20]	0.46	65	3.48 [0.21, 58.88]	0.39	95	2.31 [0.38, 13.94]	0.36	93	2.69 [0.28, 25.73]	0.39	94
**ETHNICITY**																
Asian	5	1.09 [0.95, 1.25]	0.2	0	**1.43 [1.07, 1.92]**	**0.02**	**61**	0.93 [0.67, 1.27]	0.63	0	**1.27 [1.07, 1.51]**	**<0.01**	**42**	0.74 [0.55, 1.01]	0.05	0
Non-Asian	2	2.40[0.54,10.75]	0.25	97	1.35 [1.00, 1.84]	0.05	34	3.77 [0.27, 53.30]	0.33	95	2.57 [0.54, 12.31]	0.24	91	2.76 [0.32, 24.12]	0.36	95
**DESIGN**																
Hospital	2	1.08 [0.88, 1.33]	0.44	0	**1.44 [1.07, 1.94]**	**0.02**	**18**	0.97 [0.64, 1.48]	0.88	0	1.30 [0.98, 1.71]	0.07	0	0.80 [0.54, 1.19]	0.28	0
Population	5	1.47 [0.94, 2.30]	0.09	90	**1.44 [1.05, 1.97]**	**0.02**	**61**	1.56 [0.58, 4.14]	0.38	86	**1.54 [1.04, 2.27]**	**0.03**	**77**	1.26 [0.43, 3.66]	0.67	90
**HWE**																
HWE-Yes	4	1.57 [0.90, 2.74]	0.12	92	1.49 [0.99, 2.25]	0.06	71	1.74 [0.51, 5.89]	0.38	89	**1.67 [1.00, 2.77]**	**0.05**	**83**	1.35 [0.37, 4.97]	0.65	92
HWE-No	3	1.11 [0.93, 1.32]	0.26	0	**1.41 [1.10, 1.80]**	**<0.01**	**0**	0.97 [0.66, 1.43]	0.9	0	**1.29 [1.02, 1.63]**	**0.03**	**0**	0.82 [0.57, 1.18]	0.29	0

*Population, Population controls; Hospital, Hospital controls; N, number of studies. The bold values mean the results were statistically significant*.

**Figure 2:**
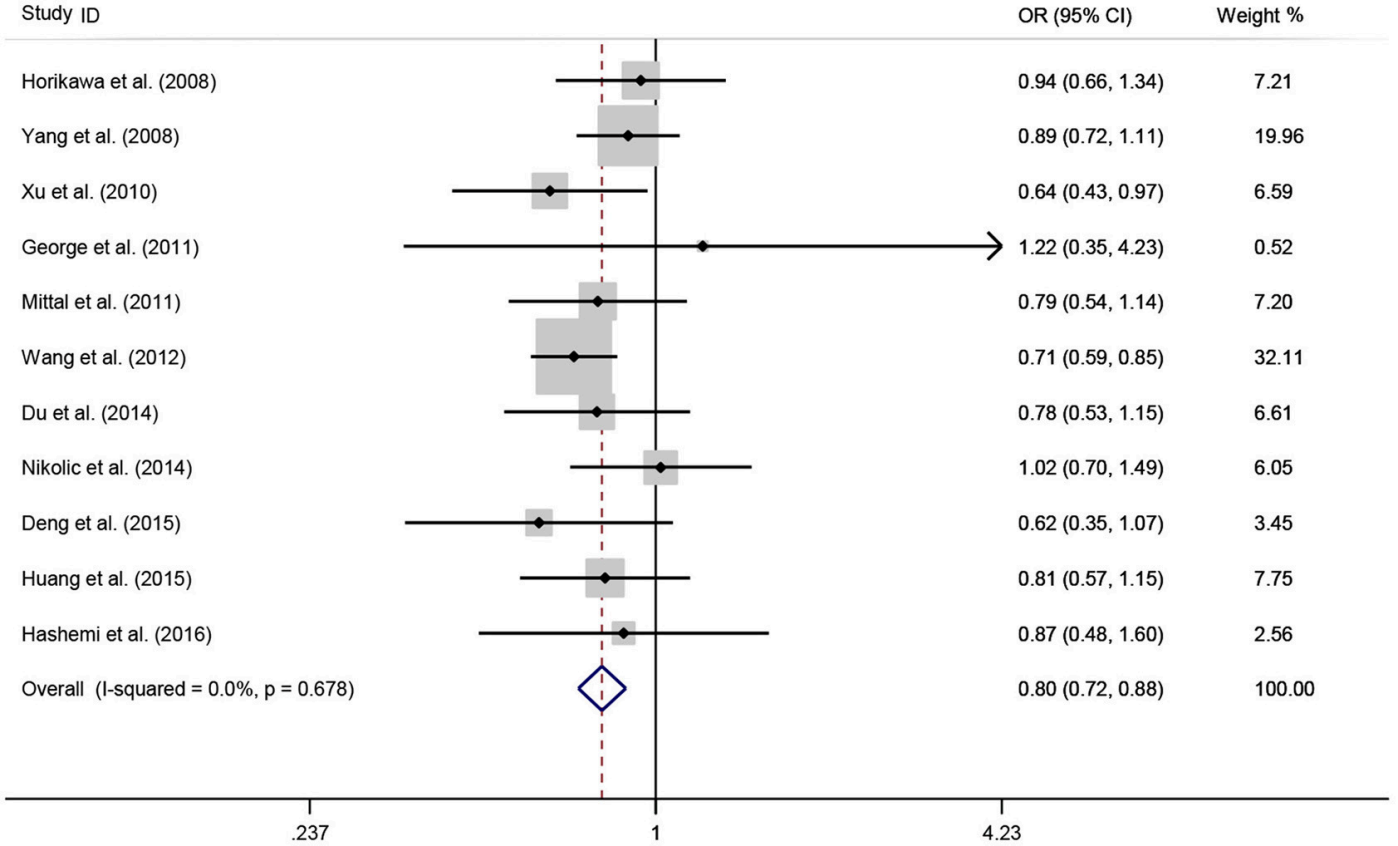
**OR and 95% CIs for the associations between microRNA-146a rs2910164 G>C polymorphism and urological cancer risk in dominant genetic model for overall populations**.

**Figure 3:**
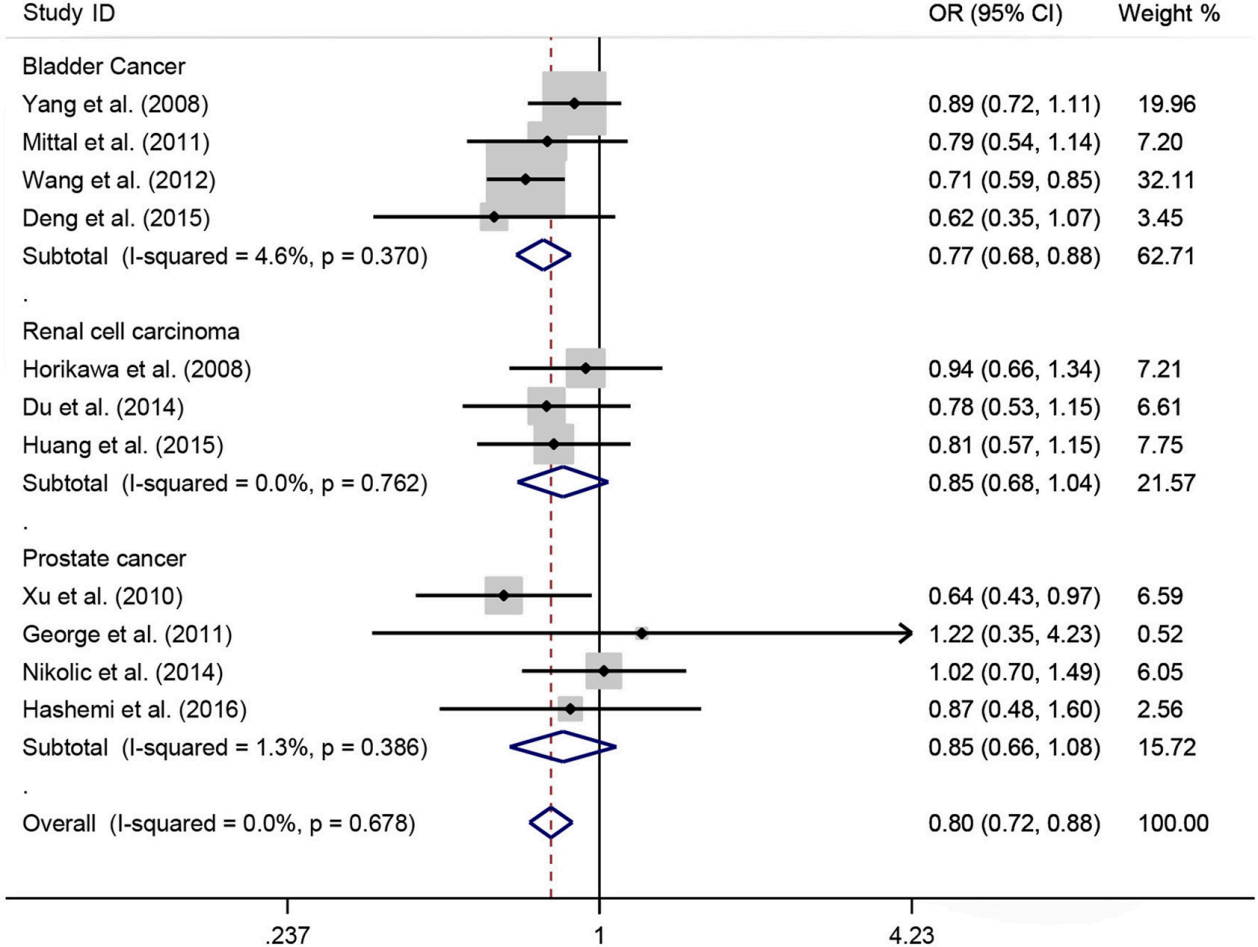
**OR and 95% CIs for the associations between microRNA-146a rs2910164 G>C polymorphism and urological cancer risk in dominant genetic model stratified by cancer type**.

**Figure 4:**
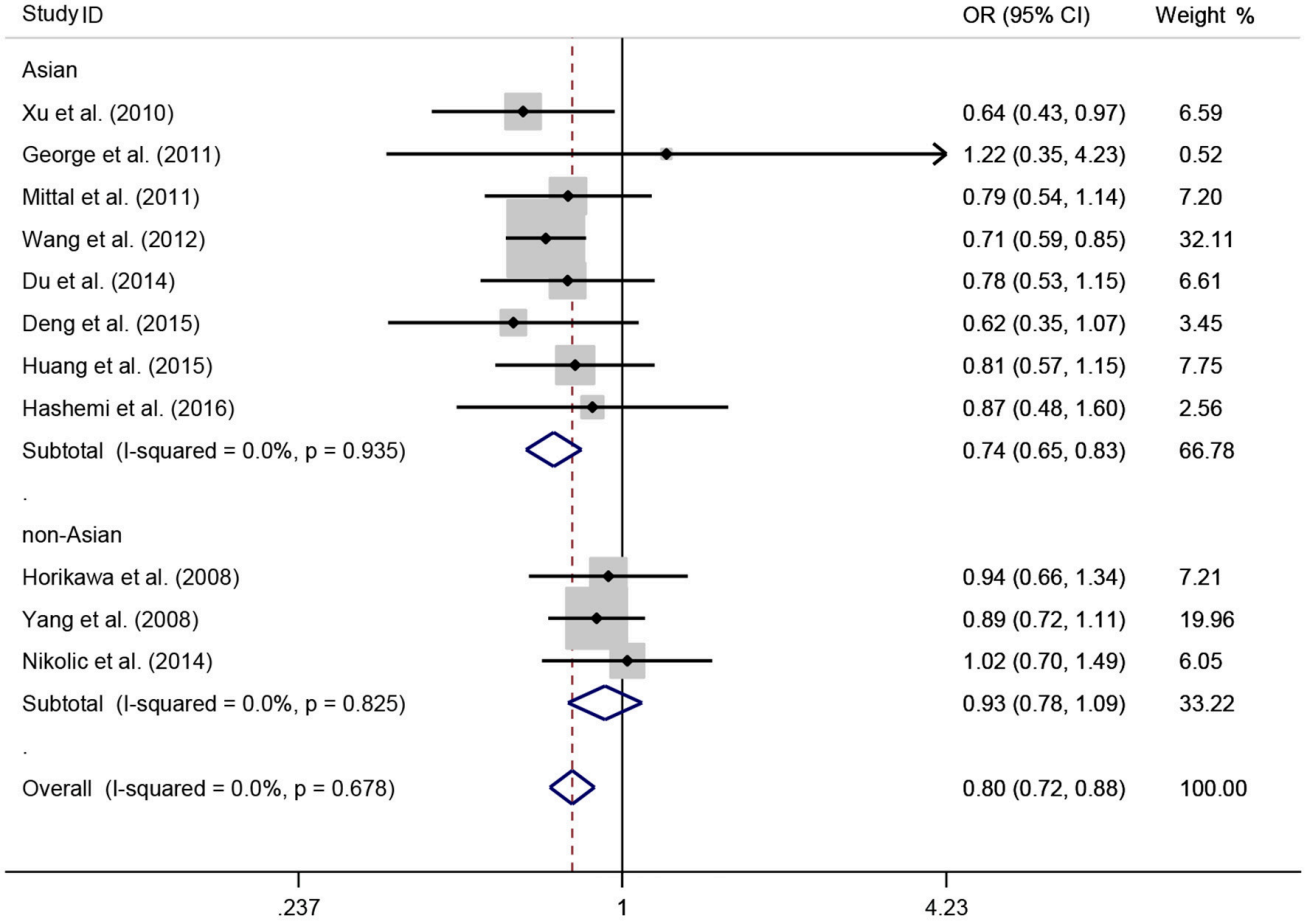
**OR and 95% CIs for the associations between microRNA-146a rs2910164 G>C polymorphism and urological cancer risk in dominant genetic model stratified by ethnicity**.

**Figure 5:**
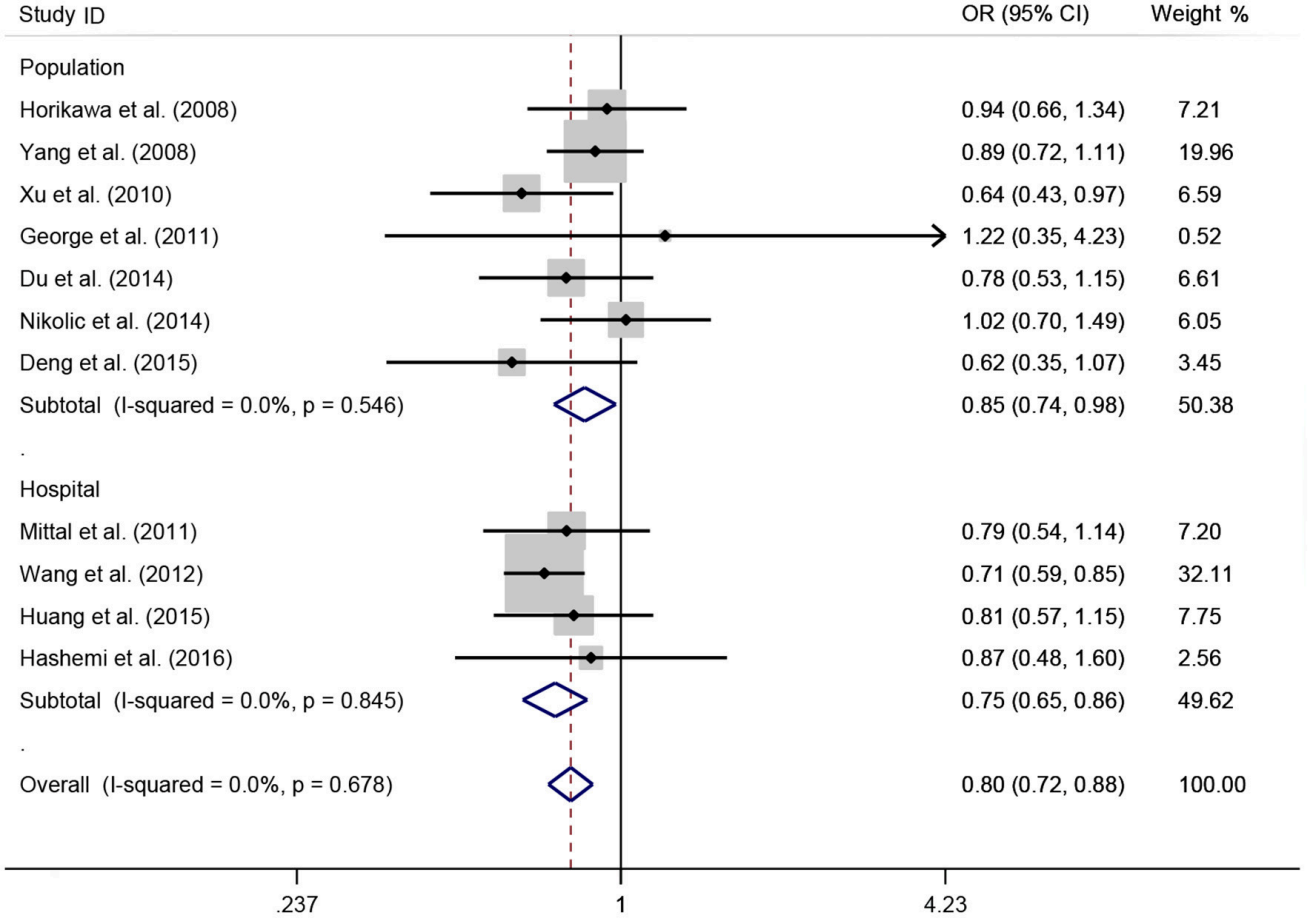
**OR and 95% CIs for the associations between microRNA-146a rs2910164 G>C polymorphism and urological cancer risk in dominant genetic model stratified by source of control**.

**Figure 6:**
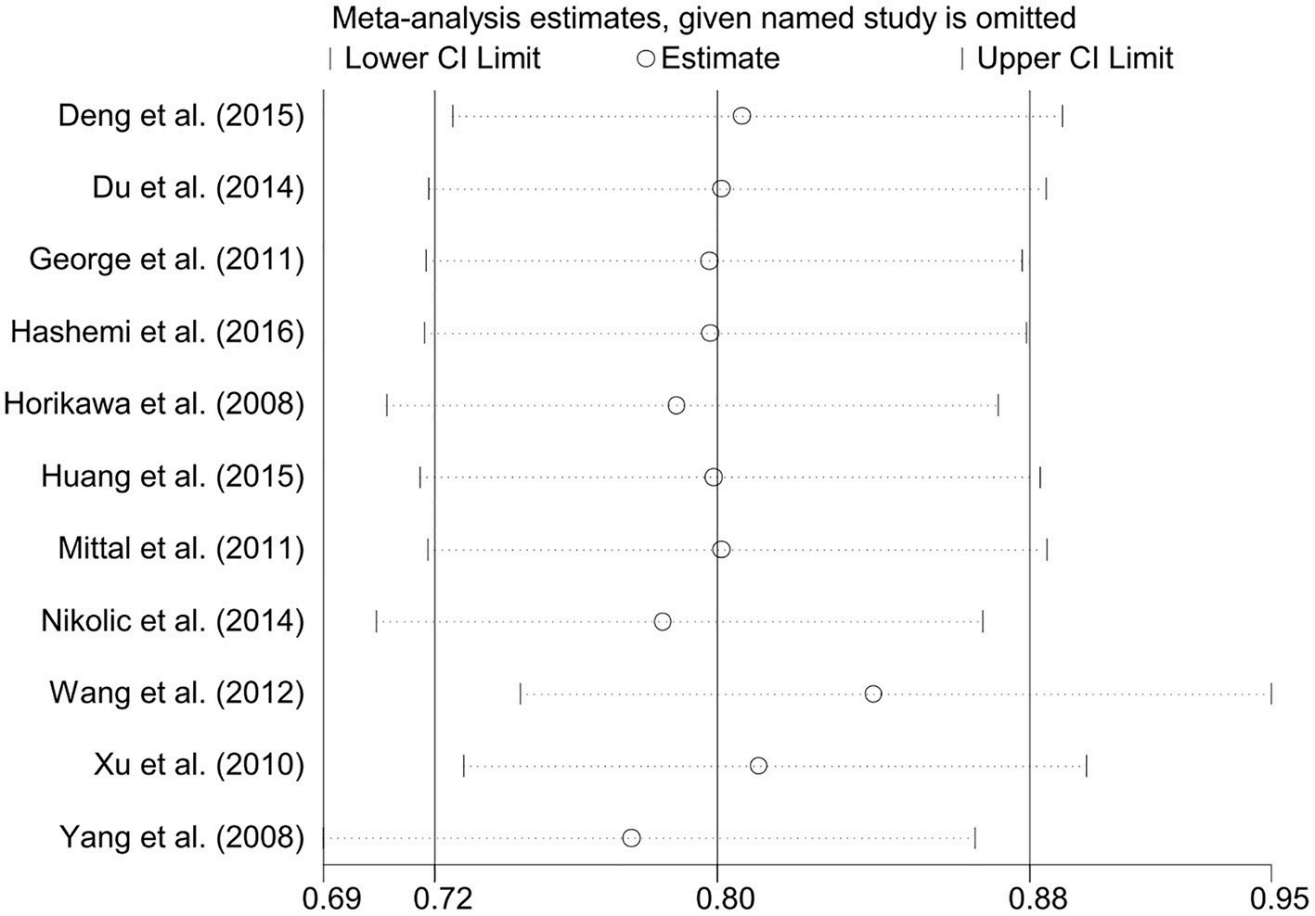
**Sensitivity analysis through the deletion of each study to reflect the individual influence on the calculated ORs in dominant genetic model of microRNA-146a rs2910164 G>C polymorphism**.

**Figure 7:**
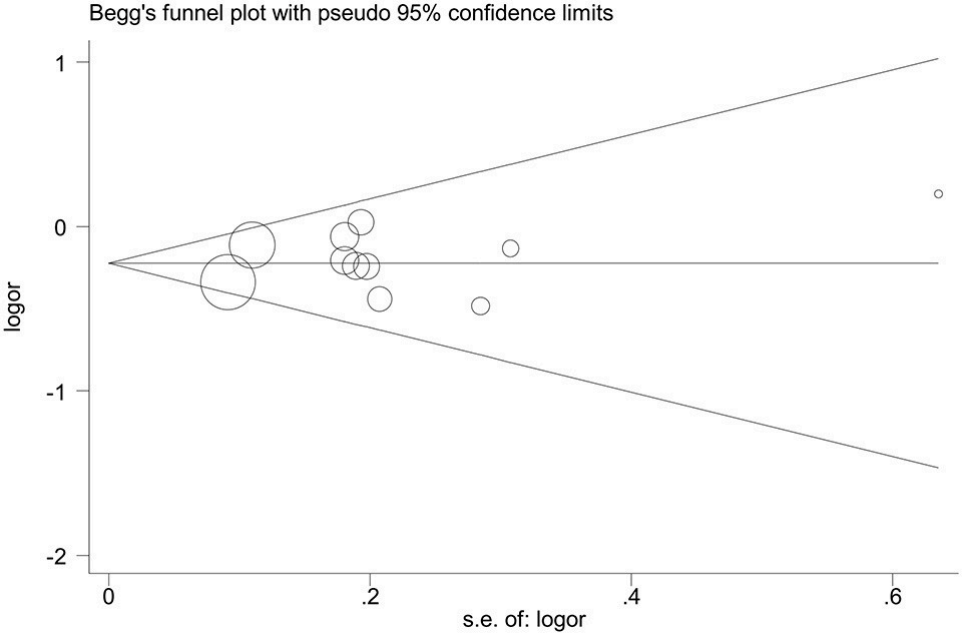
**Funnel plot analysis to detect publication bias for dominant genetic model of microRNA-146a rs2910164 G>C polymorphism. The weight of studies is presented by the size of circles**.

A total of 11 eligible studies, consisting of 3,943 cases and 4,321 controls focused on miR-146a rs2910164 G>C. The overall OR with its 95% CI revealed a significantly reduced risk of urological cancers in all the five genetic models (C vs. G: OR = 0.87, 95%CI = 0.81–0.93, *P* < 0.01, *I*^2^ = 0%; GC vs. GG: OR = 0.81, 95%CI = 0.73–0.90, *P* < 0.01, *I*^2^ = 0%; CC vs. GG: OR = 0.73, 95%CI = 0.63–0.85, *P* < 0.01, *I*^2^ = 0%; CC+GC vs. GG: OR = 0.80, 95%CI = 0.72–0.88, *P* < 0.01, *I*^2^ = 0%; CC vs. GC+GG: OR = 0.87, 95%CI = 0.77–0.98, *P* = 0.02, *I*^2^ = 0%, Figure 2; Table 3). In the tumor type stratified analysis, significant reduced cancer risk was found in four genetic models of bladder cancer (C vs. G: OR = 0.84, 95%CI = 0.77–0.92, *P* < 0.01, *I*^2^ = 0%; GC vs. GG: OR = 0.78, 95%CI = 0.68–0.89, *P* < 0.01, *I*^2^ = 0%; CC vs. GG: OR = 0.72, 95%CI = 0.59–0.87, *P* < 0.01, *I*^2^ = 0%; CC+GC vs. GG: OR = 0.77, 95%CI = 0.68–0.88, *P* < 0.01, *I*^2^ = 5%, Figure 3; Table 3). For prostate cancer and renal cell carcinoma, no diversity was detected. In subgroup analysis by ethnicity, we also observed significantly reduced cancer risks in Asian populations for all genetic models (C vs. G: OR = 0.85, 95%CI = 0.78–0.91, *P* < 0.01, *I*^2^ = 0%; GC vs. GG: OR = 0.76, 95%CI = 0.66–0.86, *P* < 0.01, *I*^2^ = 0%; CC vs. GG: OR = 0.69, 95%CI = 0.59–0.82, *P* < 0.01, *I*^2^ = 0%; CC+GC vs. GG: OR = 0.74, 95%CI = 0.65–0.83, *P* < 0.01, *I*^2^ = 0%; CC vs. GC+GG: OR = 0.85, 95%CI = 0.75–0.97, *P* = 0.01, *I*^2^ = 0%, Figure 4; Table 3). Furthermore, reduced cancer risk was also observed by the analysis restricted to HWE studies in all genetic models. And these results were consistent with analysis of the hospital control group (Figure 5). Sensitivity analysis was conducted, and no change of the result was detected (Figure 6). Meanwhile, no publication bias was observed with Egger's test (C vs. G: *P* = 0.08; GC vs. GG: *P* = 0.59; CC vs. GG: *P* = 0.07; CC+GC vs. GG: *P* = 0.54; CC vs. GC+GG: *P* = 0.18; Figure 7).

For miR-499 rs3746444 A>G polymorphism, seven studies that focused on the association of miR-499 rs3746444 A> G polymorphism and urological cancer risk involving 1,473 cases and 1,779 controls were pooled into the meta-analysis. Significant association was found in two genetic models (AG vs. AA: OR = 1.37, 95%CI = 1.18–1.60, *P* < 0.01, *I*^2^ = 49%; GG+AG vs. AA: OR = 1.43, 95%CI = 1.09–1.88, *P* < 0.01, *I*^2^ = 67%; Table 3), as well as in Asian populations (AG vs. AA: OR = 1.43, 95%CI = 1.07–1.92, *P* = 0.02, *I*^2^ = 61%; GG+AG vs. AA: OR = 1.27, 95%CI = 1.07–1.51, *P* < 0.01, *I*^2^ = 42%; Table 3). Significant association with increased urological cancer risk was also observed in AG vs. AA genetic models (OR = 1.44, 95%CI = 1.07–1.94, *P* = 0.02, *I*^2^ = 18%; Table 3) in hospital-based controls and two genetic models (AG vs. AA: OR = 1.44, 95%CI = 1.05–1.97, *P* = 0.02, *I*^2^ = 61%; GG+AG vs. AA: OR = 1.54, 95%CI = 1.04–2.27, *P* = 0.03, *I*^2^ = 77%; Table 3) in population-based control groups. Meanwhile, miR-499 rs3746444 A>G showed significant correlation with risks in prostate cancer (AG vs. AA: OR = 1.68, 95%CI = 1.17–2.41, *P* < 0.01, *I*^2^ = 58%; GG+AG vs. AA: OR = 1.45, 95%CI = 1.17–1.80, *P* < 0.01, *I*^2^ = 26%). The pooled ORs did not display any change with sensitivity analysis. Publication bias was not conducted because of the small number of studies (<10) focused on miR-499 rs3746444 A>G polymorphism.

## Discussion

Since SNPs in miRNA genes could potentially influence the miRNA biogenesis and alter target selection (Georges et al., 2007), increasing attention has been paid to evaluate the correlation between the polymorphisms in microRNAs and cancer risk (Ryan et al., 2010). Because miRNAs must correctly recognize their target sites, a SNP in microRNA may affect the process of the post-transcriptional regulation, which may cause the dysregulation of their target genes and subsequently relate to disease susceptibility. In a meta-analysis conducted by Feng et al. (2016) identified that mir-149 rs2292832 may contribute to increased susceptibility of breast cancer. Significantly increased risk between miR-146a rs2910164 and head and neck cancer (HNC) risk was observed in Caucasian population (Niu et al., 2015b). While the finding of a meta-analysis revealed that no significant association was observed between miR-149 rs2292832 and overall cancer risk, the miR-146a rs2910164 is a protective factor for bladder cancer, prostate cancer in Asians (Ma et al., 2013).

In the present meta-analysis, we discussed three SNPs in miRNAs (rs11614913, rs2910164, and rs3746444) which were considered to have certain correlation to cancer risk by pooled results from 29 eligible case-control studies including 7,927 cases and 9,092 controls. The results demonstrated that miR-146a rs2910164 G>C was a significantly reduced urological cancer risk, especially for bladder cancer, which is in agreement with the work conducted by Ma et al. (2013). It has been shown that miR-146a inhibited bladder cancer progression by targeting PTTG1, which may be the target of bladder cancer therapy (Xiang et al., 2016). Our results further confirmed that miR-146a rs2910164 G>C can reduce the risk of bladder cancer. miR-499 rs3746444 A>G polymorphism might play an important role in the development of urological cancer and might influence the risk of prostate cancer. As for miR-196a2 rs11614913 C>T, no significant association was found. However, in the subgroup analysis by cancer type, T mutation increased the risk of developing renal cell carcinoma in the CT vs. CC genetic model. Interestingly, subgroup analyses by ethnicity showed a significant association with urological cancer risk in all three polymorphisms in Asian populations. Our meta-analysis also confirmed the results of previous studies that different distribution of genotype may be a crucial risk for urological cancer susceptibility in different ethnicity (Hashemi et al., 2016). This discrepancy might be caused due to different living environment, diets, climate and lifestyles.

To our knowledge, it was the first quantitative study focused on the association between microRNA polymorphisms and urological cancer risk specially to date. The strengths our study are listed as follows: first, most of the genotype distributions in controls were consistent with HWE. Second, the relationship was analyzed by using five kinds of genetic models, and the results were statistically significant. Third, the methodological issues for meta-analysis, such as Egger's test, Begg's funnel plots and subgroup analysis were performed to ensure the stability of the results.

However, we also pay attention to the limitations in our meta-analysis. Firstly, the small sample size of studies included was still inadequate, so that the statistical power was reduced. Secondly, the effects of combination genetic and environmental factors could not be taken into account because accurate individual information was not available. Thirdly, there might exist publication bias because of the inclusion of published articles only. Finally, the diversity of genotyping methods could partly bring about the change of analyzed results.

In conclusion, the data of our meta-analysis indicate that the miR-146a rs2910164 C allele is a protective gene mutation for urological cancer, especially for bladder cancer in Asian. miR-499 rs3746444 polymorphisms may contribute to increased susceptibility of urological cancer. However, miR-196a2 rs11614913 only serve as a risk factor for renal cell carcinoma in particular in Asian populations. Further multi-centric investigation still need to confirm the relationship of these polymorphisms in microRNAs and urological cancer susceptibility.

## Author contributions

FW, C-HY, and YW conceived the study. YW, JJ, CL, and C-QY searched the databases and extracted the data. YW, HH, and HC analyzed the data. YW wrote the draft of the paper. FW and C-HY reviewed the manuscript. All the authors approved the final manuscript.

### Conflict of interest statement

The authors declare that the research was conducted in the absence of any commercial or financial relationships that could be construed as a potential conflict of interest.
